# Functional cobalt-doped hydrogel scaffold enhances concurrent vascularization and neurogenesis

**DOI:** 10.1186/s12951-025-03218-z

**Published:** 2025-04-10

**Authors:** Junqing Liu, Jun Kang, Ting Zou, Mingxin Hu, Yuchen Zhang, Shulan Lin, Ye Liang, Jialin Zhong, Yi Zhao, Xi Wei, Chengfei Zhang

**Affiliations:** 1 Hospital of Stomatology, Guanghua School of Stomatology, Sun Yat-Sen University, Guangdong Provincial Key Laboratory of Stomatology, Guangzhou, China; 2 Restorative Dental Sciences, Faculty of Dentistry, The University of Hong Kong, Hong Kong, China; 3 Shenzhen Clinical College of Stomatology, School of Stomatology, Southern Medical University, Shenzhen Stomatology Hospital (Pingshan) of Southern Medical University, Shenzhen, China; 4 Department of Obstetrics, The Third Affiliated Hospital of Zhengzhou University, Zhengzhou, China; 5 Strait Institute of Flexible Electronics (SIFE, Future Technologies), Fujian Key Laboratory of Flexible Electronics, Fujian Normal University, Fuzhou, China

**Keywords:** Hypoxia, Stem cells from apical papilla, Hydrogel, Vascularization, Neurogenesis, Tissue engineering

## Abstract

Achieving functional tissue regeneration hinges on the coordinated growth of intricate blood vessels and nerves within the defect area. However, current strategies do not offer a reliable and effective way to fulfill this critical need. To address this challenge, a three-dimensional (3D) gelatin methacryloyl–multi-walled carbon nanotube/cobalt (GelMA–MWCNTs/Co) hydrogel with controlled release of cobalt (Co) ions was developed for hypoxia-mimicking and dual beneficial effects on promoting vasculogenesis and neurogenesis. GelMA–MWCNTs/Co hydrogel exhibited sustained release of Co ions, promoting laden cell viability and long-term cell survival. GelMA–MWCNTs/Co hydrogel effectively enhanced human umbilical vein endothelial cells (HUVECs) vasculogenesis when cocultured with stem cells from apical papilla (SCAP). Moreover, this hydrogel facilitated the interaction between the pre-formed vascular and neural-like structures generated by electrical stimulation-induced SCAP (iSCAP). Furthermore, our in vivo study revealed that the GelMA–MWCNTs/Co hydrogel remarkably enhanced neovascularization and accelerated anastomosis with the host vasculature. The pre-vascularized scaffolds boosted the presence of neural differentiated SCAP in the regenerated tissue. This study provided proof of integrating functional Co ions release materials and dental-derived stem cells within a hydrogel scaffold as a promising potential for achieving simultaneous vascularization and neurogenesis.

**Graphical abstract:**

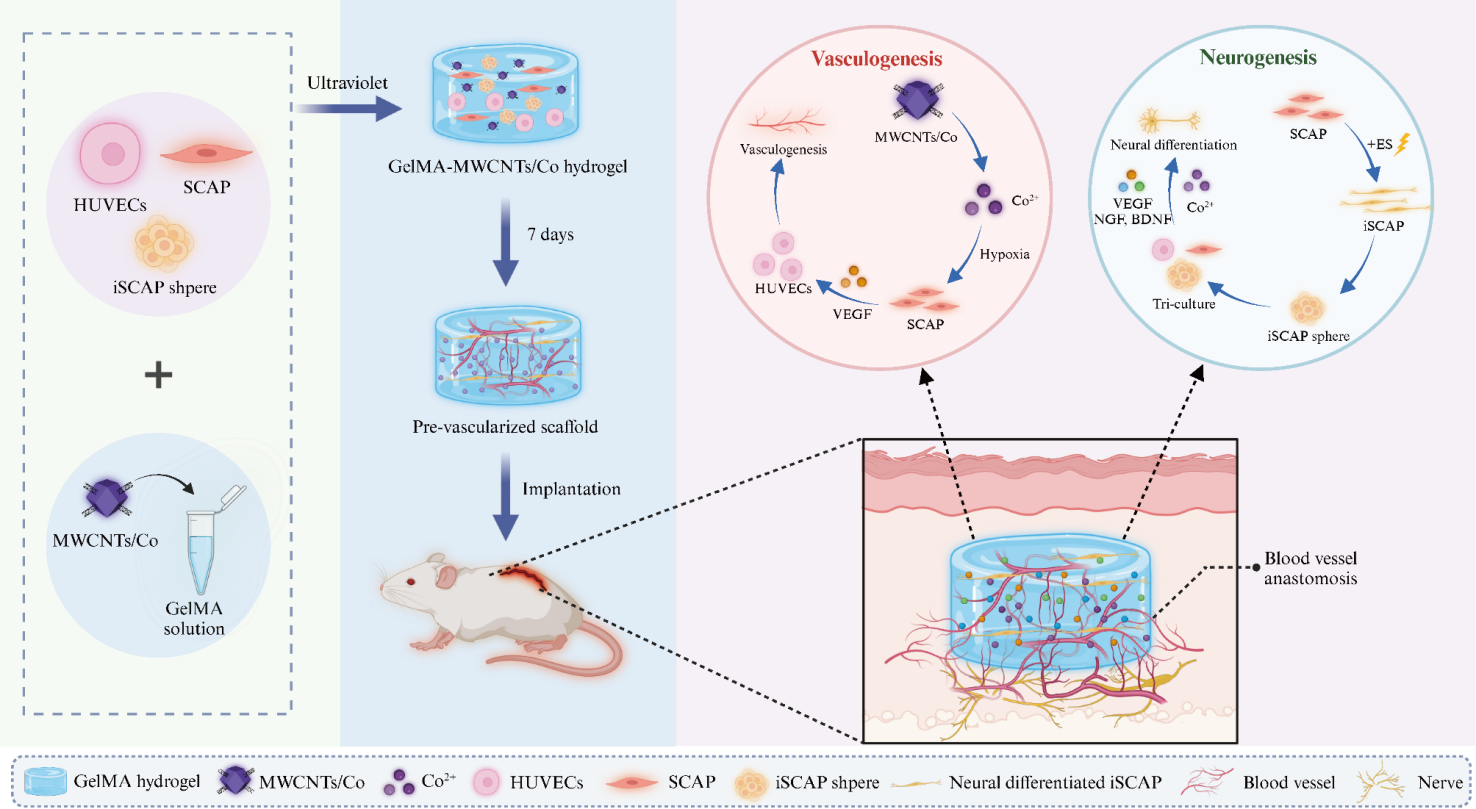

## Introduction

Tissue defects caused by traumatic injuries or tumor resection remain a serious clinical challenge. Various tissue loss can lead to vascular and neural impairment, thereby hindering the functional recovery of the affected tissues/organs. Although autologous tissue grafting is considered the most effective treatment, its application is often restricted by the limited availability of high-quality donor tissue and potential donor site morbidity. Stem cell-based tissue engineering approaches have emerged as promising alternatives for tissue regeneration to repair/restore defects [1]. These approaches involve the utilization of bioactive scaffolds to support stem cells and growth factors, facilitating the reconstruction of damaged tissues. However, a critical issue in these approaches is the difficulty of simultaneously reconstructing the blood vessels and nerves within complex defect sites. The intricate interplay between vascular and neural networks is pivotal in the regenerative process of most body tissues, as they transport essential nutrients, cytokines, and neurotransmitters [2]. At the same time, a noteworthy challenge lies in the compromised viability of cells located in the central region of the scaffold, resulting from inadequate oxygen and nutrient supply, as the natural diffusion distance of oxygen and nutrients from capillaries is limited to approximately 200 μm primarily due to slow angiogenesis [3]. Considering these factors, an effective and ideal regenerative strategy requires simultaneously promoting functional vascularization and innervation.

The engineered pre-vascularization strategy for transplanted tissues offers a promising solution. This approach entails establishing a functional vascular network within in vitro engineered tissue constructs prior to implantation. Studies have demonstrated that pre-vascularized tissue constructs exhibit improved anastomosis with the host vasculature after implantation compared to the non-vascularized one, ensuring adequate energy supply to the encapsulated cells and improving their viability [4]. In addition, there is a strong link between angiogenesis and re-innervation. Extensive studies have demonstrated that the establishment of the vascular system is closely related to nerve repair following injury [5–7]. By supplying a steady stream of blood, oxygen, and nutrients, the vascular system plays a vital role in maintaining the microenvironmental homeostasis of the nervous system [8, 9]. Moreover, vascular endothelial cells can secrete bioactive molecules that favor neurite elongation and facilitate the migration of Schwann cells [10]. Accordingly, given the superior physiological properties of pre-vascularized constructs, they are anticipated to effectively facilitate tissue regeneration by promoting complete vascularization and neurogenesis.

Hypoxia is pivotal in activating a series of angiogenesis and neurogenesis processes, primarily mediated by hypoxia-inducible factor 1α (HIF-1α) [11, 12]. As a critical mediator of hypoxia-induced response, HIF-1α plays a vital role in promoting blood vessel formation and tissue regeneration by regulating specific cytokines. For instance, vascular endothelial growth factor (VEGF), which is activated by HIF-1α, not only promotes vascular formation but also stimulates the differentiation of neural stem cells, thereby driving angiogenesis and neurogenesis [8, 12]. Furthermore, cobalt ions (Co^2+^), as the chemical hypoxia preconditioning regent, prevent the hydroxylation of HIF-1α, thereby stabilizing its expression within cells and active downstream genes, contributing to cell proliferation, differentiation, erythropoiesis, and ischemia-reperfusion processes [13]. These findings strongly support the promotion of angiogenesis through HIF-1α modulation. Thus, targeting HIF-1α is a promising strategy to induce vascularization in engineered tissues.

Metal–organic frameworks (MOFs), characterized by the advantages of abundant catalytic active sites, have been investigated for biomedical applications. Compared to pure MOFs, loading MOFs onto carbon nanotubes (CNTs) offers significant advantages. First, CNTs act as a support material that effectively prevents the aggregation of MOFs, thereby significantly enhancing their dispersion and maintaining their high specific surface area and favorable catalytic properties [14]. Second, the particle size of the loaded MOFs is typically much smaller than that of pure MOFs. This reduction in particle size not only improves dispersion in liquid media but also increases the contact area with the surrounding environment, facilitating the diffusion of ions [14, 15]. However, there have been no reports utilizing zeolitic imidazolate frameworks (ZIF-67), a cobalt-based MOF, for the in situ incorporation of MWCNTs into GelMA hydrogel to investigate its biomedical applications in vascularization and neurogenesis, particularly taking advantage of the effective release of cobalt ions. In our previous study, we fabricated the multi-walled carbon nanotubes/cobalt (MWCNTs/Co) nanocomposite, which could effectively simulate hypoxic conditions, indirectly enhancing angiogenesis in human umbilical vein endothelial cells (HUVECs) [16]. Further, we found the potential of this nanocomposite for promoting neurogenesis in the stem cells from apical papilla (SCAP) [17]. However, investigations into the effects of this hypoxia-mimicking biomaterial on the direct co-culture of HUVECs and SCAP, specifically targeting simultaneous vascularization and neurogenesis, have not been explored. In this study, we developed the three-dimensional (3D) GelMA–MWCNTs/Co hydrogel scaffold based on the Co^2+^ sustained release strategy and proposed its potential for achieving concurrent vascularization and neurogenesis through in vitro and in vivo studies. Our findings highlight the significant potential of this scaffold in promoting both vascularization and neurogenesis in the field of regenerative medicine.

## Materials and methods

### Fabrication of GelMA–MWCNTs/Co hydrogel

The MWCNTs/Co was synthesized following the procedure in a previous study [16]. Briefly, 15 mg of pretreated MWCNTs and 175 mg of polyvinylpyrrolidone were dispersed in 25 mL of methanol. Then, 498 mg of cobalt nitrate hexahydrate (Co(NO_3_)_2_·6H_2_O; J&K Scientific Ltd., Beijing, China) was added to the solution, followed by stirring for 1 h. Subsequently, 2-methylimidazole (Sigma-Aldrich, St. Louis, MO, USA) and methanol were introduced to the mixture. After 24 h, MWCNTs/ZIF-67 were generated through centrifugation, followed by washing with methanol. The nanocomposites were further heated to 300 °C in a muffle furnace for 1 h, forming MWCNTs/Co nanocomposites.

GelMA hydrogel was synthesized with slight modifications based on a previously reported method [18]. Type A porcine skin gelatin (10% w/v; Sigma-Aldrich, ) was dissolved in Dulbecco’s phosphate-buffered saline (DPBS) at 50 °C, and methacrylic anhydride (0.8 mL/g; Sigma-Aldrich) was added to the gelatin solution with 2 h stirring. Then, preheated DPBS was used to terminate the reaction, and the mixture was dialyzed against distilled water using a dialysis membrane (Spectrum Labs, Piscataway, NJ, USA) for 7 days at 40 °C, followed by lyophilization for 5 days to obtain GelMA hydrogel.

GelMA–MWCNTs/Co hydrogel was prepared as follows. The 5% w/v of GelMA was dissolved in PBS containing 0.25% w/v lithium phenyl (2,4,6-trimethylbenzoyl) phosphinate (LAP; Suzhou Intelligent Manufacturing Research Institute, Suzhou, China) photoinitiator. Then, MWCNTs/Co nanocomposites were dispersed to the GelMA solution and photocrosslinked using ultraviolet (UV) light (405 nm) for 30 s to induce gelation.

### Characterization of GelMA–MWCNTs/Co hydrogel

The morphologies of GelMA and GelMA–MWCNTs/Co hydrogels were evaluated using a Hitachi S4800 FEG SEM (Hitachi, Tokyo, Japan). Elemental analysis was performed using Energy-dispersive X-ray spectroscopy (EDX) on the SEM. To investigate the Co ion release profile, the bare MWCNTs/Co and GelMA–MWCNTs/Co hydrogel were separately immersed in α-minimal essential medium (α-MEM; Gibco-Invitrogen, Carlsbad, CA, USA) for 1, 3, 7, 10, 14, and 21 days at 37 °C. The supernatant was collected and detected using inductively coupled plasma-optical emission spectrometry (ICP-OES; SPECTRO Analytical Instruments, Kleve, Germany). The rheology experiment was conducted on the Anton Paar MCR302 rheometer (Anton Paar, Graz, Austria).

For the mechanical testing, GelMA and GelMA–MWCNTs/Co hydrogels were shaped into disks (13 mm in diameter and 7 mm in thickness). Measurements were then performed using an Instron 5942 testing machine (Instron, Norwood, MA, USA) at a speed of 1 mm/min until the samples fractured.

To evaluate the degradation rate of hydrogels, GelMA and GelMA–MWCNTs/Co hydrogels were prepared into disk shapes (13 mm in diameter and 7 mm in thickness) and immersed in PBS at 37 °C for 24 h to achieve equilibrium swelling. Subsequently, the hydrogels were transferred to PBS containing 2 U/mL of Collagenase II (EFL-Col II-DE-001, EFL) and incubated at 37 °C. At designated time points (0, 1, 2, 4, and 6 h), samples were removed and freeze-dried. The initial dry weight was recorded as W_0_. The final dry weight was recorded as W_n_ (n = predetermined time point). The mass loss was calculated using the following equation:

$$\:\text{Degradation}\:\text{ratio}\left({100\%}\right)=\frac{\text{W}_{0}-\text{W}_\text{n}}{\text{W}_{0}}\times100\%$$

### Co-culture of HUVECs with SCAP in the hydrogels

SCAP were isolated and grown following a previously published method [16]. In brief, the apical papilla of extracted human third molars was excised and digested with 3 mg/mL collagenase type I and 4 mg/mL dispase (Gibco-Invitrogen). The cells were collected after centrifugation and cultured in α-MEM, supplemented with 15% fetal bovine serum, 0.292 mg/mL glutamine, and 1% penicillin and streptomycin at 37 °C in a humidified atmosphere with 5% CO_2_. Human umbilical vein endothelial cells were obtained from ScienCell Research Laboratories (San Diego, CA, USA) and cultured in endothelial cell medium (ECM, ScienCell) at 37 ℃ with 5% CO_2_ in an incubator.

To prepare the 3D hydrogel scaffold, both HUVECs and SCAP were co-cultured. First, each cell type was individually harvested when they reached approximately 80% confluence. Then, the harvested cells were mixed in the 5% GelMA or GelMA–MWCNTs/Co prepolymer solution at a ratio of HUVECs: SCAP = 1:1, with a total cell density of 5 × 10^6^ cells/mL. The hydrogel solutions were then photocrosslinked using UV exposure for 30 s to form 3D hydrogel scaffolds.

### In vitro biocompatibility of GelMA–MWCNTs/Co hydrogel

The biocompatibility of HUVECs and SCAP cocultured in the GelMA and GelMA–MWCNTs/Co hydrogels was evaluated by live/dead/viability/cytotoxicity kit (Molecular Probes, Inc., Eugene, OR, USA) according to the manufacturerʼs instructions. After being encapsulated in the hydrogels and cocultured for 1, 3, and 7 days, the cells were stained with ethidium homodimer-1 (2 mM) and calcein acetoxymethyl (4 mM) in PBS. The fluorescently labeled cells were subsequently observed with ZEISS Confocal Microscope (LSM 900, Germany). The number of living and dead cells was counted by Fiji software [19], and the cell viability was calculated using the following equation:

$$\:\text{Cell}\:\text{viability}\left(100\%\right)=\frac{\text{living}\:\text{cell}\:\text{numbers}}{\text{total}\:\text{cell}\:\text{numbers}}\times100\%$$

The effect of the GelMA–MWCNTs/Co hydrogel on the proliferation of cocultured cells was assessed by CCK-8 assays (Dojindo, Kumamoto, Japan) according to the manufacturer’s instructions. Briefly, HUVECs and SCAP were encapsulated in the hydrogels and seeded in the 12-well plate at a total density of 5 × 10^6^ cells/mL. After coculturing for 1, 3, 5, and 7 days, the CCK-8 reagent was added to the medium. The supernatant was transferred to a 96-well plate, and the absorbance was measured at 450 nm using a SpectraMax M2 microplate reader (Molecular Devices, Sunnyvale, CA, USA).

### External electrical stimulation

The SCAP encapsulated within the GelMA or GelMA–MWCNTs/Co hydrogels were cultured in 6-well dishes with neural induction medium. The cells were then subjected to electrical pulse stimulation using a C-Dish (IonOptix, Milton, MA, USA) in combination with a pulse generator (C-Pace 100; IonOptix). The C-Dish features carbon electrodes immersed in the cell culture medium, which receive bipolar electrical stimuli from the pulse generator. A direct current electric field of 1 V/cm was applied across the carbon electrodes for a duration of 1 h each day to promote neural differentiation.

### 3D electrical stimulation-induced SCAP (iSCAP) spheres

After 7 days of electrical stimulation, iSCAP were collected by lysing the hydrogels using GelMA Lysis Buffer (EFL-GM-LS-001, Suzhou Intelligent Manufacturing Research Institute, Suzhou, China). To fabricate microtissue spheres of iSCAP, 256-well agarose micro-molds (MicroTissues, Inc, Sharon, MA, USA) were utilized following the manufacturer’s instructions. Briefly, 500 µL of molten agarose was pipetted into each well of the micro-mold. After the agarose was gelled, the micro-mold was carefully flexed to remove the 3D Petri Dish, which was then transferred to a 12-well plate. Subsequently, 2.5 mL of cell culture medium was added to the 12-well plate and allowed to equilibrate for at least 1 h. The culture medium was then removed, and iSCAP cell suspensions (2.56 × 10^5^ cells/190 µL) were transferred to the 3D Petri Dish and cultured for 3 days at 37 °C in a 5% CO_2_ atmosphere. Afterward, the iSCAP spheres were collected and utilized for further coculture experiments.

### Enzyme-linked immunosorbent assay (ELISA)

To quantify VEGF protein secretion from SCAP, the cell culture supernatants were collected, and the secreted VEGF was quantified using the Human VEGF DuoSet ELISA Kit (R&D Systems, Minnesota, USA) according to the manufacturer’s instructions.

### Quantitative real-time PCR (qRT-PCR)

To evaluate the level of VEGF mRNA in the SCAP and HUVECs co-culture system, as well as the expression of neural-specific markers, including βΙΙΙ-tubulin (Tuj1), microtubule associated protein (MAP2), neuronal nuclear protein (NeuN), nerve growth factor (NGF), brain-derived neurotrophic factor (BDNF) in iSCAP following growth with HUVECs, qRT-PCR was performed. Total RNA was extracted with the RNeasy Mini kit (Qiagen, Hilden, Germany) and then reverse-transcribed to cDNA using the PrimeScriptTM (RR036A, Takara, Shiga, Japan). qRT-PCR was performed using a TB Green Premix Ex Taq (RR420A, Takara) on a QuantStudio 6 Flex (Applied Biosystems, Grand Island, NY, USA). Relative gene expression was standardized by β-actin. The primers for VEGF, Tuj1, MAP2, NeuN, and β-actin were obtained from Beijing Liuhe Huada Gene Technology Co., Ltd (Beijing, China), and the sequences are shown in Table 1.

**Table 1:** Primers used for the qRT-PCR test

Primer name	Sequences(5’-3’)
VEGF	F: TTGCCTTGCTGCTCTACCTCCA R: GATGGCAGTAGCTGCGCTGATA
Tuj1	F: CTCAGGGGCCTTTGGACATC R: CAGGCAGTCGCAGTTTTCAC
MAP2	F: GGCATTGAAGAATGGCAGAT R: CCCTGTATGGGAATCCATTG
NeuN	F: TGGCATGACCCTGTACACAC R: TGCTTCTCTGTAGGGTCGGA
NGF	F: TGTGGGTTGGGGATAAGACCA R: GCTGTCAACGGGATTTGGGT
BDNF	F: TAACGGCGGCAGACAAAAAGA R: TGCACTTGGTCTCGTAGAAGTAT
β-actin	F: CACCATTGGCAATGAGCGGTTC R: AGGTCTTTGCGGATGTCCACGT

### Western blot analysis

HUVECs and SCAP were cocultured in GelMA and GelMA–MWCNTs/Co hydrogel with a total cell density of 5 × 10^6^ cells/mL. After 24, 48, and 72 h, the hydrogel with cells was collected and soaked in lysis buffer, which included the RIPA and protease inhibitor cocktail (Thermo Fisher Scientific, Waltham, MA, USA), at 4 °C for 1 h. The BCA protein assay kit (Thermo Fisher Scientific) was then used to calculate the protein concentration. Equivalent amounts of protein from each lysate were separated by SDS-PAGE gels and transferred onto PVDF membranes (Millipore, Billerica, MA, USA). Then, membranes were blocked with 5% skim milk, followed by incubation with primary antibodies anti-HIF-1α (BD Biosciences, New Jersey, USA) and anti-β-actin (Santa Cruz Biotechnology) at 4 °C overnight. The membranes were incubated with HRP-conjugated anti-mouse (Cell Signaling Technology) second antibody for 1 h. The immunoreactive bands were detected following incubation with ECL (Thermo Fisher Scientific) and visualized by an iBright FL 1500 Imaging System (Thermo Fisher Scientific).

### Immunofluorescence (IF)

To observe the vasculogenesis process during the coculture of HUVECs and SCAP, the cells were cultured in GelMA and GelMA–MWCNTs/Co hydrogels for 7 and 14 days. To visualize the process of vascularization and neural differentiation, as well as their integration during the coculture of iSCAP and HUVECs within the hydrogels, the cells were cultured for 7 days. Afterward, the cells were fixed in 4% paraformaldehyde for 30 min at room temperature and then blocked in a serum-blocking buffer containing 5% serum and 0.3% Triton™ X-100 for 1 h. Subsequently, the samples were incubated overnight at 4 °C with the following primary antibodies: anti-CD31 (#3528, 1:400, Cell Signaling Technology, Danvers, MA, USA), anti-SM22α (ab14106, 1:400, Abcam, Cambridge, MA, USA), anti-beta III Tubulin (Tuj1) antibody (NB100-1612, 1:300, Novus Biologicals, Littleton, CO, USA). After primary antibody incubation, the cells were incubated with secondary antibodies: Alexa Fluor 488 goat anti-mouse IgG (#4408, 1:500, Cell Signaling Technology), Alexa Fluor 594 anti-rabbit IgG (#8889, 1:500, Cell Signaling Technology), Alexa Fluor 488 goat anti-chicken IgY (ab150173, 1:500, Abcam), or Alexa Fluor 594 goat anti-mouse IgG (#8890, 1:500, Cell Signaling Technology) at room temperature for 1 h, followed by DAPI (Thermo Fisher Scientific) staining. The fluorescent images were acquired using the ZEISS Confocal Microscope (LSM 900, Carl Zeiss, Germany). The average vessel length and total number of vessel junctions were measured using AngioTool [20]. The neurite length was measured using the Fiji software.

### In vivo implantation of cell-loaded GelMA–MWCNTs/Co hydrogel

All experimental animal procedures were approved by the Committee on the Use of Live Animals in Teaching and Research of the University of Hong Kong (CULATR number: #5303-20). A total of twelve 6-week-old female CB-17 SCID mice were used in this study, and all surgical procedures were performed following the relevant guidelines and regulations. The mice were divided into the following four groups: GelMA-ES: HUVECs + SCAP + iSCAP (without ES) sphere in GelMA; GelMA + ES: HUVECs + SCAP + iSCAP (with ES) sphere in GelMA; GelMA/Co-ES: HUVECs + SCAP + iSCAP (without ES) sphere in GelMA–MWCNTs/Co; GelMA/Co + ES: HUVECs + SCAP + iSCAP (with ES) sphere in GelMA–MWCNTs/Co. 3 × 6 mm cylindrical hydrogel samples loaded with different cell combinations were cultured in media for 7 days before implantation. Surgical procedures were performed under anesthesia using ketamine (90 mg/kg) and xylazine (10 mg/kg) administered via intraperitoneal injection. Small subcutaneous pockets were created through a dorsal skin incision, and four hydrogel samples were implanted into the pockets on the bilateral sides of the wound. Meloxicam (1–2 mg/kg) was injected for three days after surgery to alleviate post-surgery pain. The mice were housed together. No signs of pain were observed throughout the monitoring period. At 4 weeks post-implantation, the animals were euthanized. The whole regenerated tissue specimens containing the cell-loaded hydrogels and surrounding tissue were harvested. The excised samples were fixed in 4% paraformaldehyde for 24 h at room temperature and then embedded in paraffin for histology and immunohistochemistry analysis.

### Hematoxylin and eosin (H&E) and immunohistochemical (IHC) staining

H&E and immunohistochemical staining were performed on 5 μm thick tissue sections. For H&E staining, the paraffin slides were deparaffinized in xylene (twice, 3 min each time) and rehydrated in ethanol (twice in 100% ethanol, 3 min each time; once in 95% and 70% ethanol, 3 min each time). The sections were then incubated in hematoxylin for 3 min and then rinsed with running tap water for 3 min. Subsequently, the sections were immersed in 1% acid alcohol (1% hydrochloric acid in 70% alcohol) for 1 s and washed with tap water for 3 min. Slides were then immersed in a bluing reagent (1% ammonia solution) for 1 min, followed by washing with running tap water for 3 min. After that, slides were rinsed in eosin solution for 1 min. The sections underwent dehydration before mounting with Permount mounting solution (Thermo Fisher Scientific) and were observed by a microscope (Nikon, Tokyo, Japan). The lumens containing intraluminal red blood cells were quantified as perfused blood vessels, while lumens without red blood cells were considered non-perfused blood vessels. The quantitative analysis of both total blood vessels and perfused blood vessels was conducted by counting five observed areas using Fiji software [19].

For immunohistochemical staining, the paraffin slides were deparaffinized and rehydrated, followed by antigen retrieval using Target Retrieval Solution (Citrate pH 6.0, DAKO, Santa Clara, CA, USA) at 95 °C for 40 min. Then, the staining was performed using Rabbit Specific HRP/DAB Detection IHC Kit (Abcam, Cambridge, MA, USA) according to the manufacturer’s instructions. After blocking with Protein Block Solution at room temperature for 10 min, the slices were incubated with the primary antibody of human-specific anti-CD31 antibody (ab32457, 1:200, Abcam) and anti-Tuj1 antibody (NB100-1612, 1:200, Novus Biologicals) overnight at 4 °C. The secondary antibody used was Biotinylated Goat Anti-Polyvalent, which was provided by the Dako HRP/DAB Detection IHC Kit. Then, the streptavidin peroxidase was applied and incubated for 10 min at room temperature. After that, the bound complex was visualized using DAB and counterstained with hematoxylin. Images were captured using a light microscope, and quantitative analysis was carried out by counting the number of positive cells in five observed areas with Fiji software.

### Immunofluorescent analysis of the tissue sections

Paraffin-embedded sections were subjected to immunofluorescence analysis, following the same procedures as the IHC staining. Briefly, the sections were deparaffinization, rehydration, and antigen retrieval. Subsequently, the sections were incubated with a 5% serum-blocking buffer for 1 h at room temperature, followed by overnight incubation at 4 °C with the primary antibodies: human-specific anti-CD31 antibody (ab32457, 1:200, Abcam) and anti-Tuj1 antibody (NB100-1612, 1:200, Novus Biologicals). Afterward, the sections were incubated with the secondary antibodies: Alexa Fluor 488 anti-rabbit IgG (#4412, 1:200, Cell Signaling Technology) and Alexa Fluor 488 goat anti-chicken IgY (ab150173, 1:200, Abcam) for 1 h at room temperature. Actin was stained using Actin-Tracker Red-Rhodamine (1:500, Biyuntian, Shanghai, China), while nuclei were stained and mounted using Mounting Medium with DAPI (ab104139, Abcam). Fluorescence was observed using the ZEISS Confocal Microscope (LSM 900).

### Statistical analysis

All quantitative data were presented as mean ± standard deviation (SD) for *n* ≥ 3. Statistical significance was carried out using one-way or two-way analysis of variance (ANOVA). (*p < 0.05; **p < 0.01, ***p < 0.001, ****p < 0.001).

## Results

### Synthesis and characterization of GelMA and GelMA–MWCNTs/Co hydrogels

**Fig. 1:**
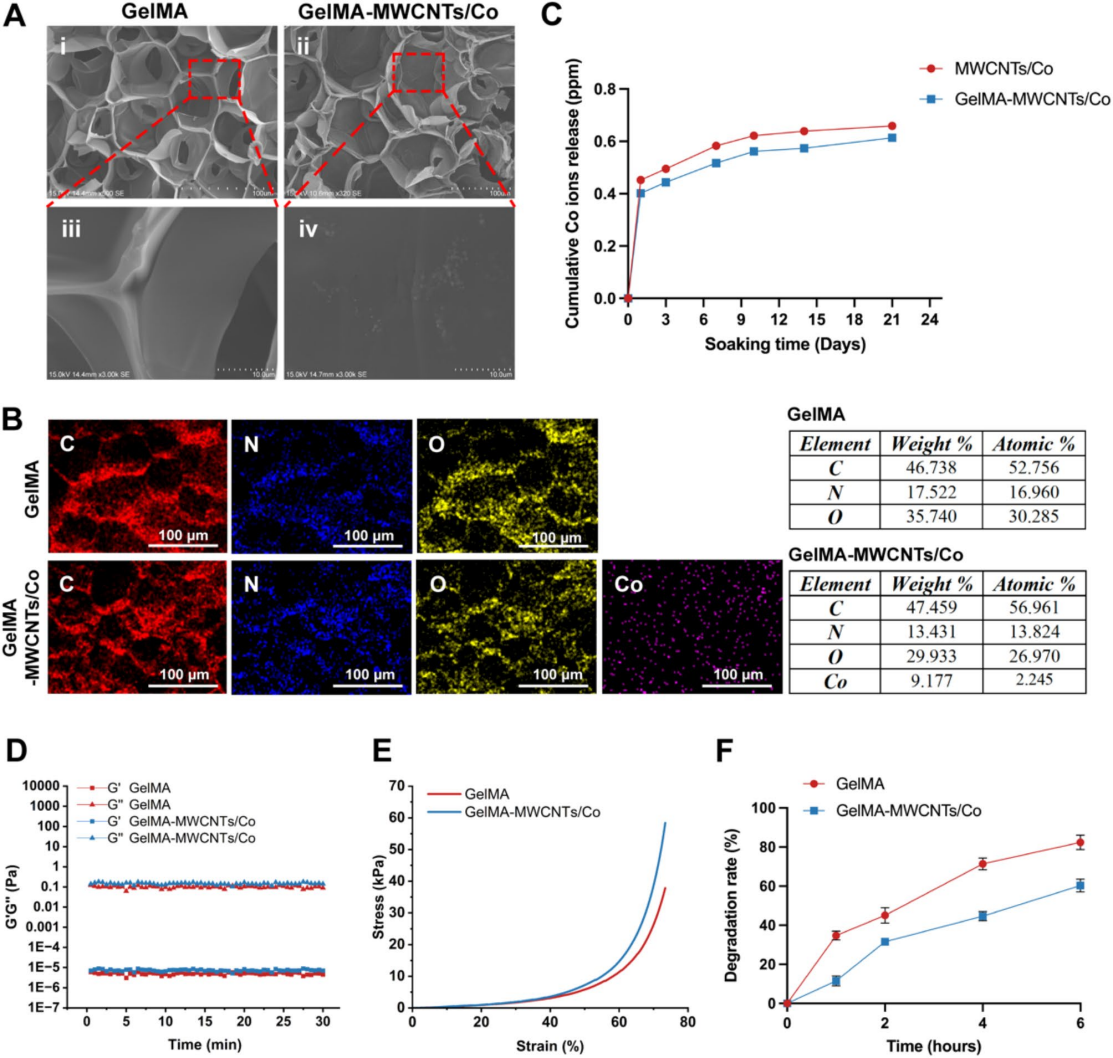
Characterization of the GelMA and GelMA–MWCNTs/Co hydrogels. (**A**) SEM images of GelMA and GelMA–MWCNTs/Co hydrogels. (**B**) EDX elemental mapping of GelMA and GelMA–MWCNTs/Co (C: Carbon, N: Nitrogen, O: Oxygen, Co: Cobalt). (**C**) Release of Co ions from the bare MWCNTs/Co and GelMA–MWCNTs/Co hydrogels in the medium. (**D**) Storage modulus (G′) and loss modulus (G″), (**E**) Compressive stress-strain curves, and (**F**) In vitro degradation ratio of GelMA and GelMA–MWCNTs/Co hydrogels (*n* = 4)

The morphologies of the GelMA and GelMA–MWCNTs/Co hydrogels were evaluated under SEM. As depicted in Fig. 1A, both hydrogels exhibited characteristic alveolate structures. The GelMA–MWCNTs/Co hydrogel showed the presence of MWCNTs/Co composites on its surface. The detailed elemental mapping by EDX analysis confirmed the homogeneous distribution of Co ions within the GelMA–MWCNTs/Co hydrogel (Fig. 1B). To mimic physiological conditions, the release of Co ions from the bare MWCNTs/Co and the GelMA–MWCNTs/Co hydrogels was quantified in a medium containing fetal bovine serum (FBS). As shown in Fig. 1C, both groups exhibited an initial burst release of Co ions on the first day. Afterward, the GelMA–MWCNTs/Co hydrogel demonstrated a significantly lower and more controlled release of Co ions than the bare MWCNTs/Co. The rheological characteristics of GelMA and GelMA–MWCNTs/Co hydrogels were assessed to understand their viscoelastic behavior. As illustrated in Fig. 1D, both hydrogels displayed similar nonlinear rheological behaviors, meanwhile, the GelMA–MWCNTs/Co hydrogels exhibited higher storage modulus (G′) and loss modulus (G″) compared to the GelMA hydrogel. The compressive strain − stress curves were obtained and combined in Fig. 1E. A distinct trend can be observed, showing that the incorporation of MWCNTs/Co into the GelMA hydrogel markedly enhances its mechanical strength. To evaluate the in vitro biodegradation behavior of the hydrogels, Collagenase II was employed to accelerate the degradation process, and the weight changes were monitored (Fig. 1F). Our results indicated that the GelMA–MWCNTs/Co hydrogel exhibited slower degradation rates compared to the GelMA hydrogel. After 6 h of enzymatic degradation, the residual mass of GelMA was approximately 18%, while the GelMA–MWCNTs/Co hydrogel remained about 41%.

### In vitro biocompatibility of 3D GelMA–MWCNTs/Co hydrogel

**Fig. 2:**
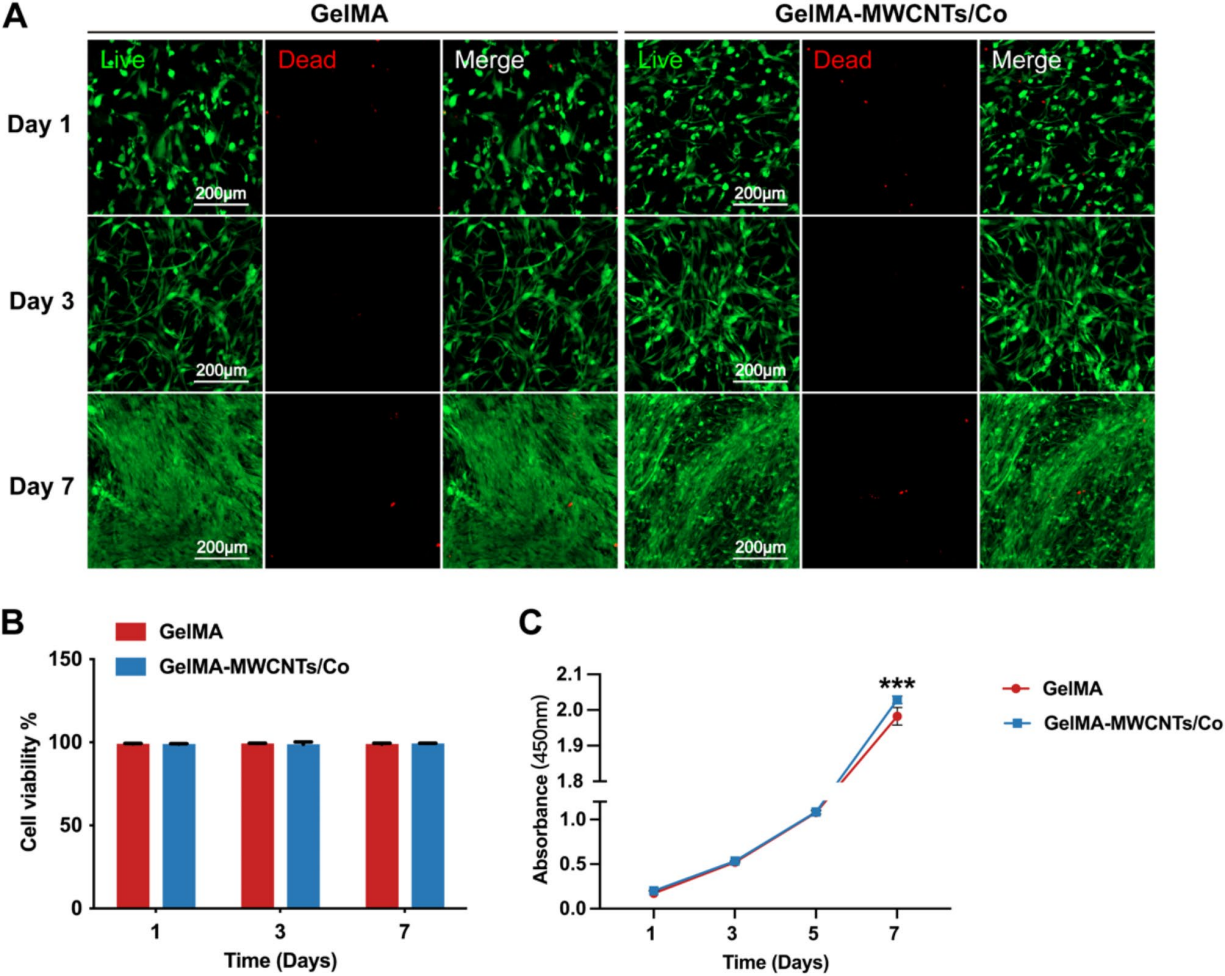
Biocompatibility of GelMA and GelMA–MWCNTs/Co hydrogels on HUVECs and SCAP encapsulation. (**A**) Fluorescence images of live/dead staining of HUVECs and SCAP coculture within the 3D hydrogels after cultured for 1, 3, and 7 days (live cells: green; dead cells: red). (**B**) Quantitative analysis of cell viability for HUVECs and SCAP in hydrogels. (**C**) CCK-8 assay for cell proliferation of HUVECs and SCAP in the hydrogels. (∗*p* < 0.05, ∗∗*p* < 0.01. ∗∗∗*p* < 0.001, *n* = 3)

Our previous study showed that MWCNTs/Co nanocomposites at 50 µg/mL enhanced SCAP viability [16]. In the current study, we explored the cellular activity within a coculture system of HUVECs and SCAP embedded in a 3D GelMA–MWCNTs/Co hydrogel. The encapsulated cells within the 3D microenvironment exhibited morphological changes from the first day of culture. Following a 7-day culture period in both GelMA and GelMA–MWCNTs/Co hydrogels, live/dead staining revealed high cell viability for HUVECs and SCAP. The number of living cells notably increased, and there was apparent morphological spreading in the culture over time, indicating cell migration and distribution throughout the entire scaffold (Fig. 2A). Quantitative analysis of cell viability showed no significant difference between the GelMA and GelMA–MWCNTs/Co groups (Fig. 2B), demonstrating that the GelMA–MWCNTs/Co hydrogel maintains comparable biocompatibility, suitable for further experiments.

The cell proliferation within the GelMA–MWCNTs/Co hydrogel was evaluated using the CCK8 assay, focusing on the coculture of HUVECs and SCAP. The proliferation rates of cells from day 1 to day 5 showed no significant difference between the GelMA and GelMA–MWCNTs/Co groups. However, on day 7, a significant increase in proliferation was observed in the GelMA–MWCNTs/Co group (Fig. 2C). This finding suggested that the GelMA–MWCNTs/Co hydrogel not only effectively supported cell adhesion but also enhanced the proliferation of both HUVECs and SCAP.

### GelMA–MWCNTs/Co hydrogel on the vasculogenic activity of HUVECs

**Fig. 3:**
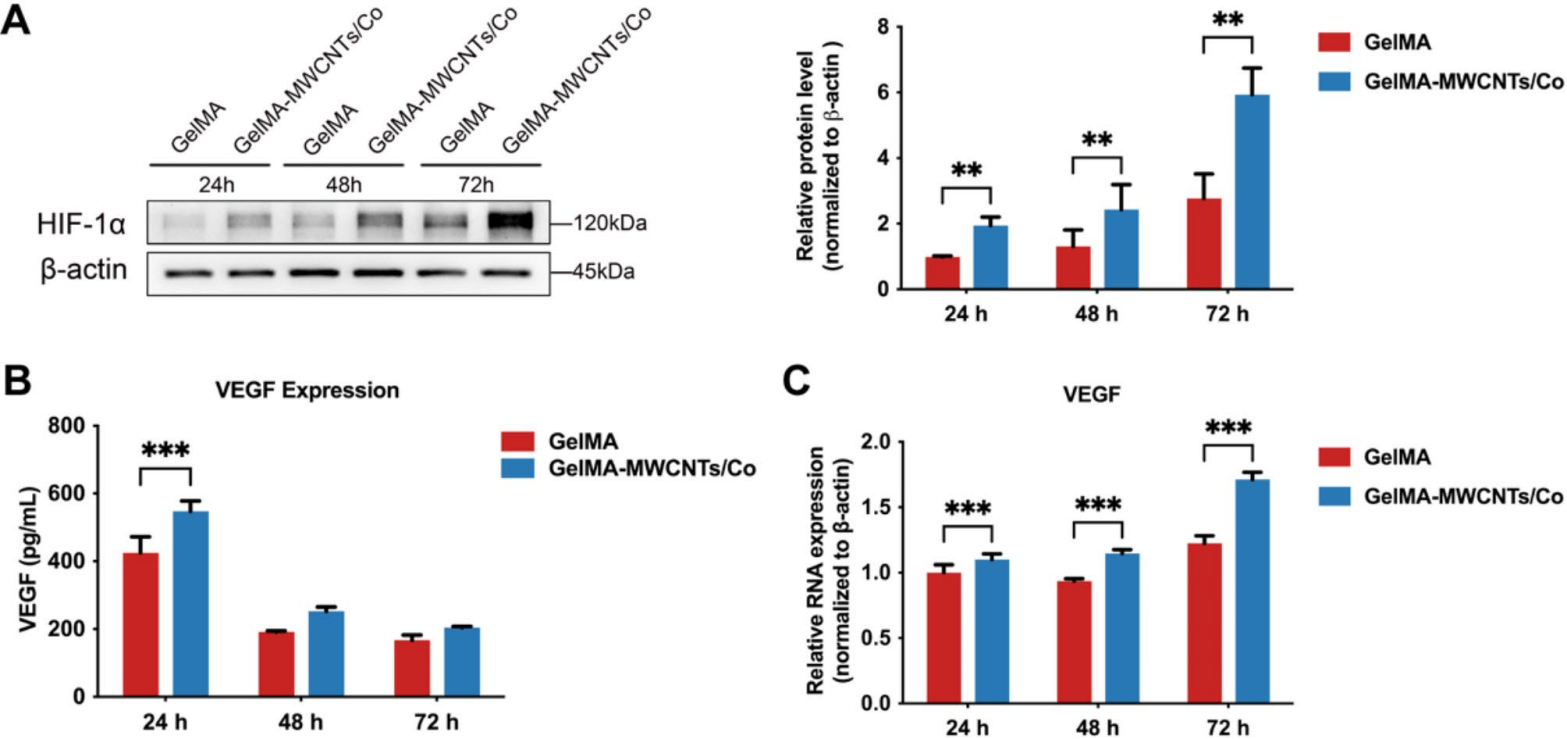
GelMA–MWCNTs/Co hydrogel on HIF-1α and VEGF expression. (**A**) Protein expression of HIF-1α in cocultured SCAP and HUVECs after growing in GelMA and GelMA–MWCNTs/Co hydrogel for 24, 48, and 72 h, determined by Western blot. (**B**) VEGF secretion levels from cocultured SCAP and HUVECs at different time intervals. (**C**) Gene expression levels of VEGF in cocultured cells at different time points. (∗*p* < 0.05, ∗∗*p* < 0.01. ∗∗∗*p* < 0.001, *n* = 3)

Based on our findings, the GelMA–MWCNTs/Co hydrogel could control the release of Co ions. However, it remains unknown whether this hydrogel can successfully mimic hypoxia and subsequently activate HIF-1α, leading to VEGF secretion when cocultured cells are grown within it. Therefore, to further explore the efficacy of the GelMA–MWCNTs/Co hydrogel in activating hypoxic-related processes in SCAP and promoting vasculogenesis in the 3D coculture system, the expression levels of HIF-1α and VEGF were evaluated using western blot, ELISA, and RT-qPCR. The western blot analysis indicated a significant elevation in HIF-1α protein expression within the GelMA–MWCNTs/Co hydrogel compared to the GelMA hydrogel at 24, 48, and 72 h, with a time-dependent increase observed in both groups (Fig. 3A). These results validate the property of GelMA–MWCNTs/Co hydrogel to enhance HIF-1α protein expression through its controlled Co ions release. ELISA results revealed that VEGF expression was significantly higher in the GelMA–MWCNTs/Co hydrogel than in the GelMA hydrogel at 24 h. However, a subsequent decrease in VEGF levels was noticed in both groups, possibly due to VEGF consumption by HUVECs for migration and vascular structure formation (Fig. 3B). To further investigate whether the lack of difference in VEGF concentration between the GelMA and GelMA–MWCNTs/Co groups at 48 and 72 h, as well as the decrease in VEGF levels in both groups, was indeed due to VEGF consumption by HUVECs, we conducted RT-qPCR to assess intracellular VEGF expression levels. The results demonstrated a time-dependent upregulation of VEGF gene expression in both groups, with the GelMA–MWCNTs/Co group exhibiting significantly higher levels compared to the GelMA group (Fig. 3C). The collective evidence from this study indicated that the GelMA–MWCNTs/Co hydrogel, designed to mimic hypoxic conditions, effectively stimulated VEGF secretion by activating HIF-1α within the coculture system, thereby promoting vascular formation.

### GelMA–MWCNTs/Co hydrogel accelerated vessel-like network formation in vitro

**Fig. 4:**
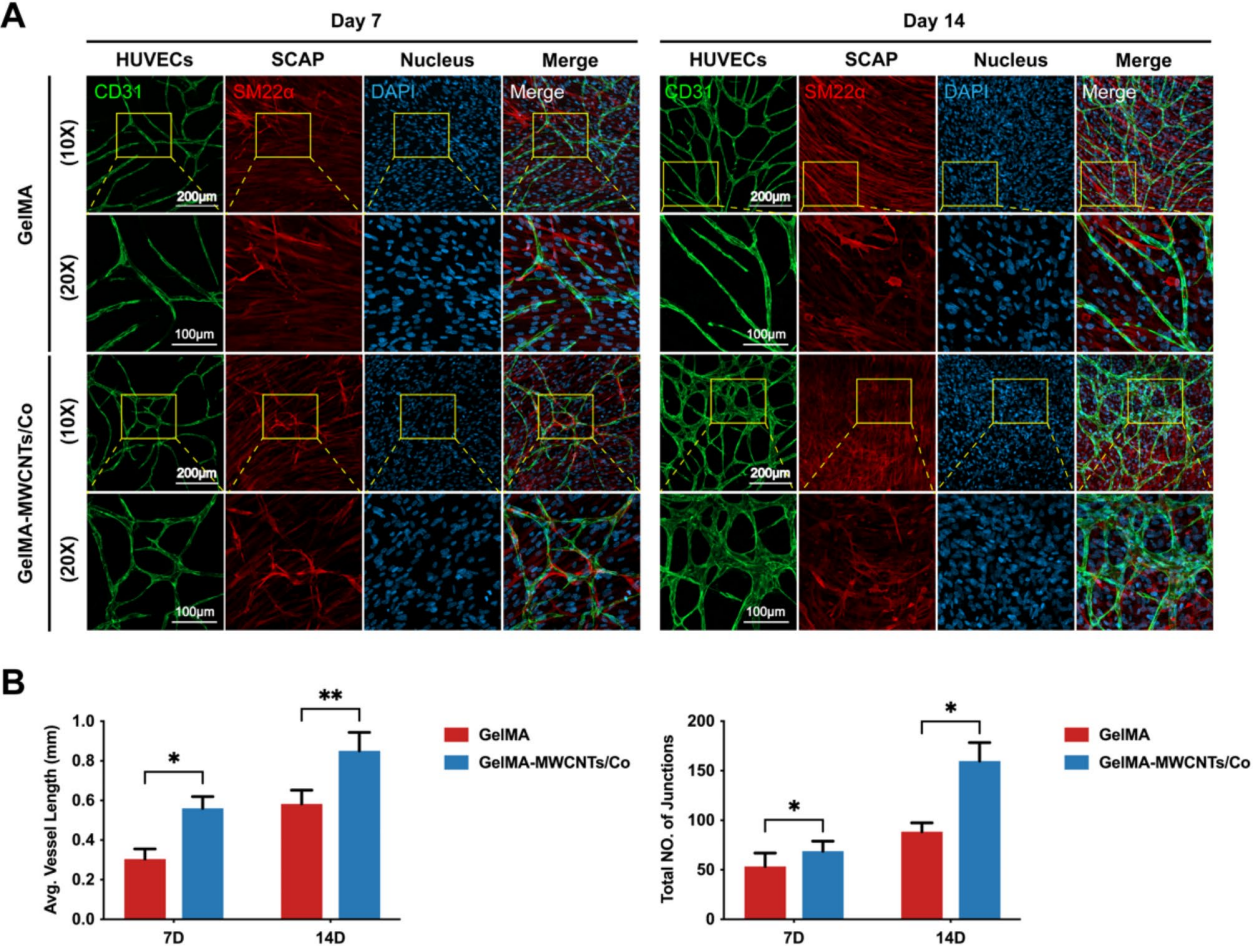
In vitro vessel-like network formation in GelMA–MWCNTs/Co hydrogel. (**A**) Representative confocal images of HUVECs (CD31: green) and SCAP (SM22α: red) cocultured in the 3D GelMA and GelMA–MWCNTs/Co hydrogels for 7 and 14 d. (Nucleus: blue). Magnification of the yellow boxed area was shown in the lower panel. (**B**) Quantitive analysis of the average vessel length and total number of vessel junctions in the hydrogel scaffolds at the different time points. (∗*p* < 0.05, ∗∗*p* < 0.01. ∗∗∗*p* < 0.001, *n* = 3)

Our previous study demonstrated that MWCNTs/Co could effectively enhance the angiogenic potential of SCAP and indirectly promote the angiogenesis of HUVECs in the 2D culture [16]. However, the angiogenic potential of direct co-culture HUVECs and SCAP has not been thoroughly explored. To address this part, HUVECs and SCAP were directly cocultured within the 3D GelMA–MWCNTs/Co hydrogel to examine vascular formation in a closely mimicked physiological environment. We monitored the spontaneous organization of these cells into vessel-like structures over the course of 14 days. By day 7, the formation of such structures was evident within both GelMA and GelMA–MWCNTs/Co scaffolds. However, the vessel-like networks within the GelMA–MWCNTs/Co hydrogel were noticeably more complex, characterized by increased vessel length and junction density (Fig. 4A-B). By day 14, these characteristics were even more pronounced, suggesting progressive maturation of the vascular network. Interestingly, during the maturation of the forming vessel network, SCAP were positively immunostained with SM22α, a specific maker of smooth muscle cells. These cells were found to be closely associated with the vessel structures, surrounding and wrapping around the vessel walls (Fig. 4A).

**Fig. 5:**
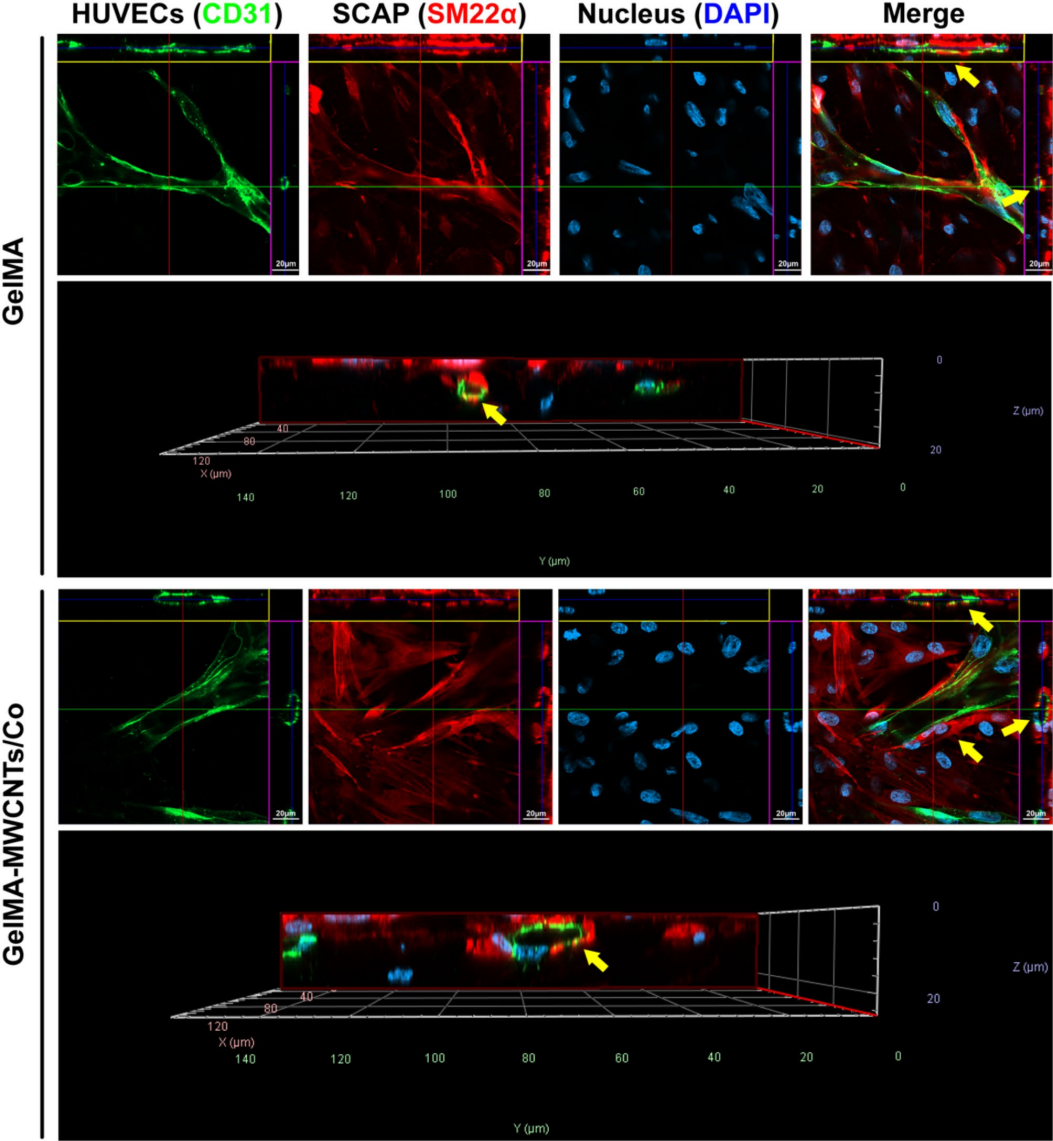
Representative orthogonal 3D-projection images of the vessel-like networks in GelMA and GelMA–MWCNTs/Co hydrogels. Yellow arrows indicate the 3D vessel lumen formation. (HUVECs: green, SCAP: red, Nucleus: blue)

Orthogonal 3D-projection images further confirmed this structural arrangement and revealed the development of luminal structures, hinting at the potential for blood perfusion (Fig. 5). These findings strongly suggested the potential functional blood perfusion following implantation, the feasibility of SCAP to differentiate into vascular smooth muscle cells, and the assured involvement of SCAP in vessel stabilization and maturation.

### Triculture of iSCAP, HUVECs, and SCAP to generate neurovascular structure

**Fig. 6:**
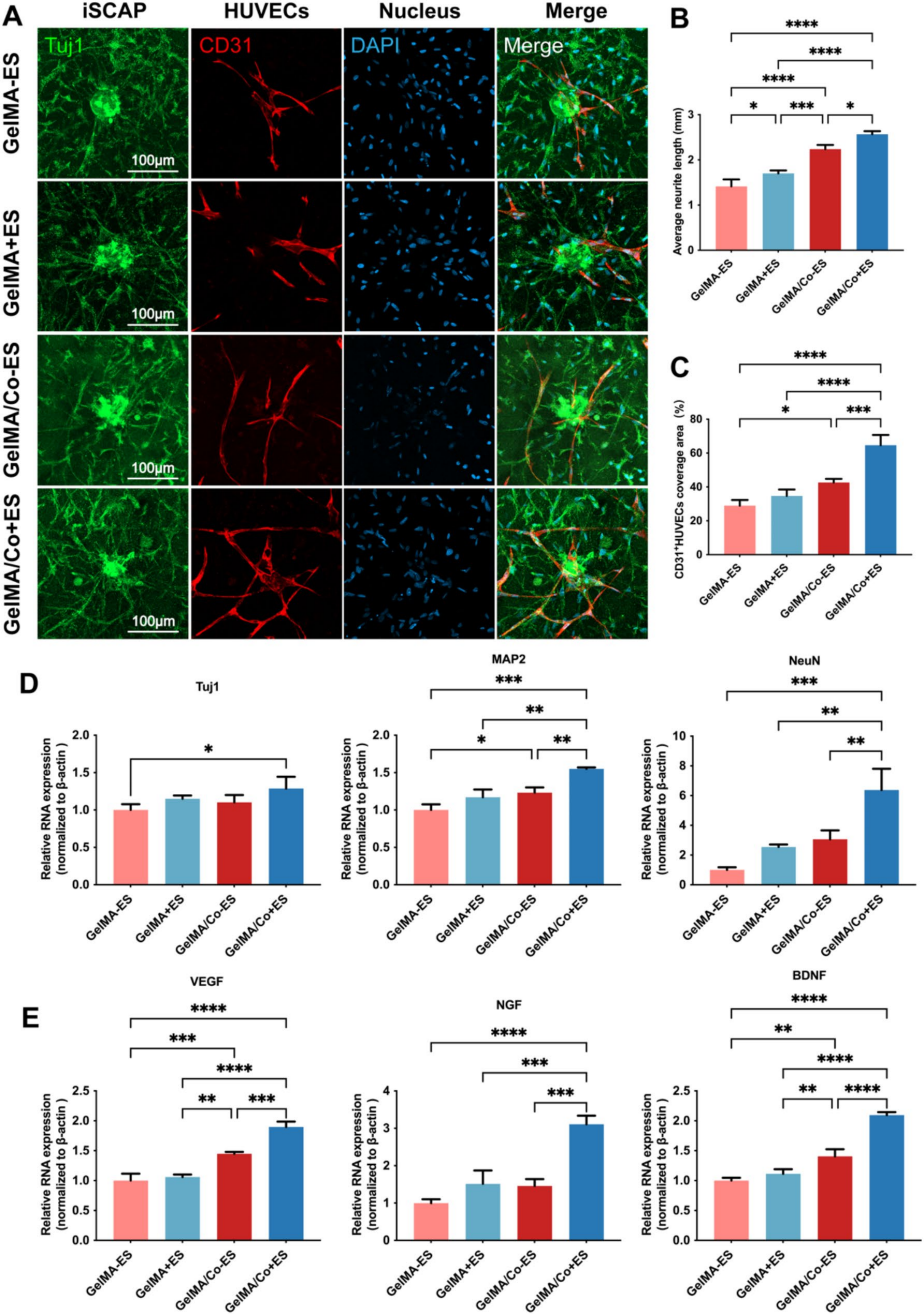
Triculture of iSCAP, HUVECs, and SCAP for generating neurovascular structures. (**A**) Representative immunofluorescence images of iSCAP (Tuj1: green), HUVECs (CD31: red), and nuclei (DAPI: blue) within 3D hydrogel on day 7. (**B-C**) Quantitative analysis of the average neurite length (**B**) and the coverage area of CD31 + positive HUVECs on the sprouting cells derived from the iSCAP microsphere (**C**). (**D**) Quantification of neural-related gene Tuj1, MAP2, and NeuN mRNA expression levels in different groups. (**E**) Quantification of angiogenic growth factor VEGF and neurotrophic factors NGF and BDNF mRNA expression levels in different groups. (∗*p* < 0.05, ∗∗*p* < 0.01. ∗∗∗*p* < 0.001, *n* = 3)

In order to investigate the synergistic growth of neovascular and neurally differentiated iSCAP, iSCAP microspheres were co-cultured with HUVECs and SCAP in different hydrogels for 7 days. Four groups were established: GelMA-ES: HUVECs + SCAP + iSCAP (without ES) sphere in GelMA; GelMA + ES: HUVECs + SCAP + iSCAP (with ES) sphere in GelMA; GelMA/Co-ES: HUVECs + SCAP + iSCAP (without ES) sphere in GelMA–MWCNTs/Co; GelMA/Co + ES: HUVECs + SCAP + iSCAP (with ES) sphere in GelMA–MWCNTs/Co. The results releaved that in both the GelMA-ES and GelMA + ES groups, there was limited cell sprouting from the iSCAP microspheres, and a few neurite-like structures were observed. However, when the iSCAP microspheres were encapsulated in hydrogels containing MWCNTs/Co (GelMA/Co-ES and GelMA/Co + ES groups), a notable enhancement in the outgrowth and extension of neurite-like structures was observed compared to the other two groups (Fig. 6A). Quantitative analysis further substantiated these observations, revealing that, particularly when coupled with electrical stimulation, the average length of the neurite-like extensions in the GelMA/Co + ES group was significantly longer than that in the GelMA/Co-ES group (Fig. 6B).

Interestingly, a similar growth pattern was observed in the vascular structures by HUVECs. For GelMA-ES and GelMA + ES groups, only a few tubular structures were formed. In contrast, cultures in MWCNTs/Co containing GelMA hydrogel exhibited a significant augmentation in vessel-like structure development, regardless of whether ES was applied to the iSCAP or not (Fig. 6A). Furthermore, the coverage area of HUVECs (human CD 31^+^) on the sprouting cells derived from the iSCAP microspheres was substantially greater in the GelMA/Co + ES group compared to GelMA/Co-ES (Fig. 6C). These findings strongly suggested synergistic interactions between the iSCAP-derived structures and the vasculature, which were further boosted by ES. The integrative crosstalk may be critical for neurovascular tissue engineering.

To further confirm the effect of the tri-culture system within GelMA–MWCNTs/Co hydrogel on neural differentiation at the genetic level, an RT-qPCR assay was employed to analyze neural gene expression after coculture. Compared to the GelMA-ES group, gene expression levels of neural markers Tuj1, MAP2, and NeuN in iSCAP were significantly upregulated in the GelMA/Co + ES hydrogel. Additionally, even though the iSCAP of both groups were already subjected to electrical stimulation, the expression levels of MAP2 and NeuN were higher in the MWCNTs/Co containing GelMA than in the pure GelMA hydrogel (Fig. 6D).

To further explore the interactions between neovascularization and neural differentiation in the triculture system, the expression levels of key angiogenic growth factor VEGF and the neurotrophic factors NGF and BDNF were assessed using RT-qPCR. Remarkably, the expression level of the VEGF gene was significantly upregulated in the GelMA/Co-ES and GelMA/Co + ES groups compared to the GelMA-ES and GelMA + ES groups. The most significant elevation in VEGF expression was detected in SCAP exposed to ES prior to being cultured in the GelMA–MWCNTs/Co hydrogel (Fig. 6E). This finding was consistent with our observation of the prominent vessel-like structures, particularly in the GelMA/Co + ES group. Interestingly, similar expression patterns were observed in the expression of NGF and BDNF, which are pivotal neurotrophic factors known for their roles in neuronal growth and nerve regeneration. Only when ES-induced iSCAP were cultured in the GelMA–MWCNTs/Co hydrogel did the degree of neural differentiation and neurotrophic factors, NGF and BDNF, reach their peak expression among all the groups (Fig. 6E).

### GelMA–MWCNTs/Co hydrogel accelerated angiogenesis and neurogenesis in vivo

**Fig. 7:**
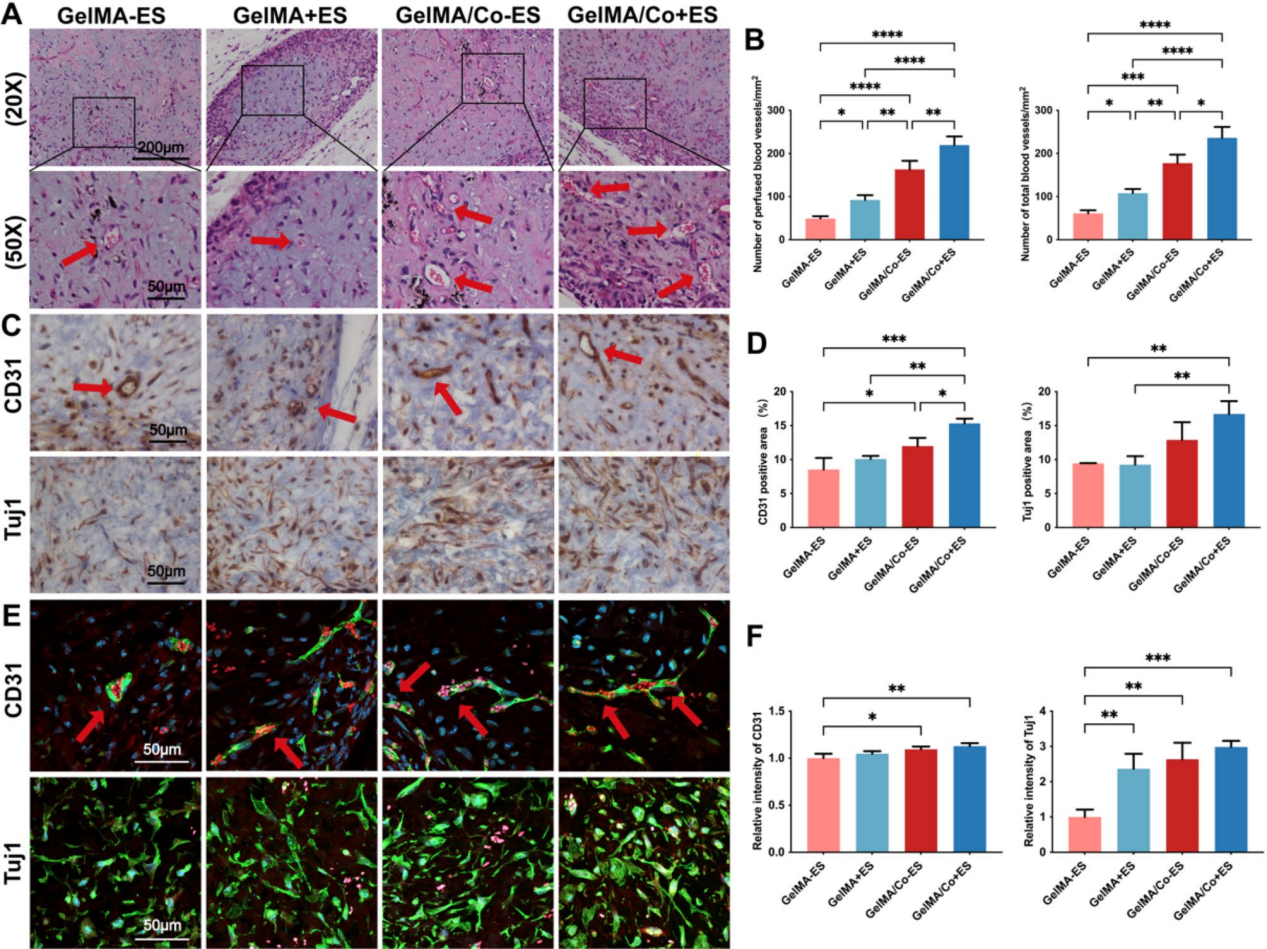
Pre-vascularized engineered GelMA–MWCNTs/Co hydrogel scaffold promoted angiogenesis and neurogenesis in vivo. (**A**) H&E staining for the 4-week post-implanted hydrogel scaffolds and surrounding tissues. Magnification of the black boxed area was shown in the lower panel, and red arrows indicated the blood vessels perfused with red blood cells. (**B**) Quantitative analysis of the number of perfused blood vessels and number of total blood vessels in different groups. (**C**) IHC staining for neovascularization (CD31) and neural differentiation (Tuj1) of different hydrogels. Red arrows indicated the human CD31-positive lumen structures. (**D**) Quantitative analysis of the CD31 and Tuj1 stained-positive area in hydrogels. (**E**) Immunofluorescence staining of CD31 (green), Tuj1 (green), Rhodamine (red), DAPI (blue). Red arrows indicated the human CD31-positive lumen structures. (**F**) Semi-quantification of positively stained cells. (∗*p* < 0.05, ∗∗*p* < 0.01. ∗∗∗*p* < 0.001, *n* = 3)

The above in vitro findings suggested that the GelMA–MWCNTs/Co hydrogel exhibited favorable cellular biocompatibility and desirable pre-vasculogenic and pre-neurogenic differentiation effects, indicating its potential for neurovascular tissue regeneration. To explore the in vivo efficacy of pre-vascularized engineered GelMA–MWCNTs/Co hydrogel scaffold in promoting angiogenesis and neurogenesis, a model of tissue formation was developed. The iSCAP microspheres with or without ES, HUVECs, and SCAP were co-cultured in either GelMA or GelMA–MWCNTs/Co hydrogels for a period of 7 days. Then, the constructs were implanted subcutaneously into SCID mice and examined after four weeks. H&E staining revealed a complete replacement of hydrogel with connective tissue and anastomosis of hydrogel-generated blood vessels with the host’s vasculature. Microvessels within the newly regenerated tissue were perfused with murine red blood cells, evidencing successful anastomosis between the implanted blood vessels and host vessels (Fig. 7A). This perfusion is a critical step towards achieving functional vascularization within the engineered tissue. Notably, the GelMA/Co + ES group displayed superior vascular integration, with a significantly higher density of perfused blood vessels within the implantation. Quantitative analysis corroborated this observation, indicating a higher vessel count and perfusion within the GelMA/Co + ES group compared to the GelMA/Co-ES, GelMA + ES, and GelMA-ES groups (Fig. 7B). These in vivo results were consistent with our in vitro vessel formation findings, where HUVECs in the GelMA/Co + ES group exhibited the most robust formation of vessel-like networks.

IHC analysis for human CD31 confirmed extensive microvessel formation in the GelMA/Co + ES group compared to the other three groups. Furthermore, the inclusion of MWCNTs/Co in the GelMA hydrogels was associated with an increased presence of Tuj1(+) positive cells, particularly pronounced in the GelMA/Co + ES group, suggesting enhanced iSCAP neural differentiation possibly due to the effects of VEGF (Fig. 7C-D). Histologically, immunofluorescence analysis further proved that the lumen structures of blood vessels in the implanted tissue were positively stained for human CD31 (Fig. 7E), indicating that the implanted HUVECs contributed to blood vessel formation in vivo. Additionally, increased intensity of CD31 and Tuj1 was observed in the GelMA/Co + ES group (Fig. 7E-F), suggesting that this group has the superior potential to promote blood vessel formation and enhance neural differentiation in vivo.

## Discussion

In the field of regenerative medicine, various materials are utilized for vascular grafts and nerve guidance conduits, chosen for their favorable biocompatibility and mechanical properties. However, their effectiveness in promoting cell adhesion and supporting nerve regeneration varies significantly. For instance, while polylactic acid demonstrates good biocompatibility, its inherent rigidity may impede the growth and migration of nerve cells [21]. In contrast, polyurethane, with its superior elasticity and flexibility, more closely mimics the physiological environment, thereby facilitating nerve regeneration [22]. Recent studies on cobalt-based biomaterials have primarily focused on promoting vascularization and osteogenesis, with less emphasis on their potential for simultaneously supporting vascularization and neurogenesis. Therefore, the application potential of the GelMA–MWCNTs/Co scaffold in both nerve and vascular repair warrants thorough exploration.

The GelMA hydrogel, known for its resemblance to the gelatin-based extracellular matrix (ECM), has been proven to exhibit natural biocompatibility [23]. Its 3D structure effectively emulates the in vivo cellular microenvironment, making it an indispensable material in the field of regenerative medicine and tissue engineering [24]. Our previous studies have demonstrated that GelMA hydrogel possesses excellent biocompatibility with dental stem cells, promoting their attachment, proliferation, and differentiation [17, 25, 26]. The current study incorporated cobalt ions into the GelMA hydrogel as a functional activator to mimic hypoxic conditions, taking advantage of its unique biological effects. Our results suggested a controlled release of Co^2+^ from the GelMA–MWCNTs/Co hydrogel (Fig. 1C). The limited diffusion of Co ions through the GelMA matrix could be attributed to two main factors. First, the potential for electrostatic interactions between the positively charged Co ions and the negatively charged GelMA hydrogel could lead to a higher binding affinity of Co ions within the GelMA matrix. This affinity may result in a reduced cumulative release of Co ions compared to the bare MWCNTs/Co. Secondly, the limited access of water molecules by GelMA may impede the hydrolysis of MWCNTs/Co in GelMA, further decreasing the release rate of Co ions. Based on these findings, we have determined that incorporating MWCNTs/Co into GelMA hydrogel allowed for a controlled release of ions, thus effectively mitigating the premature and excessive release of Co ions. The majority release of Co ions occurred during the initial 7-day period, coinciding with the period of hypoxic preconditioning before transplantation. This controlled release profile is advantageous, as the minimal release of Co ions following subsequent transplantation helped to prevent the worsening of the hypoxic environment in the newly transplanted tissue. Therefore, compared to bare MWCNTs/Co, the GelMA hydrogel not only provided structural support for cells but also played a crucial role in establishing a delivery system for controlled ion release.

Rheological measurements showed the values of storage modulus (G′) and loss modulus (G″) for both hydrogels. The incorporation of MWCNTs/Co resulted in an increase in the storage modulus (G′), and a similar trend was observed in the loss modulus (G″). This indicates the development of weak percolation networks of MWCNTs/Co within the hydrogel matrix. The mechanical properties of a hydrogel are critical in determining its feasibility and functionality. For successful nerve regeneration, a scaffold must provide adequate mechanical support for neurite outgrowth and create neurogenic niches that facilitate neural signal transmission among neuronal cells along axons [27]. Additionally, the mechanical behavior of scaffolds influences the fate of cells in various tissue engineering applications. For instance, stem cells grown on stiffer substrates tend to differentiate preferentially into bone cells, while those on moderately stiff substrates may differentiate into muscle cells, and cells on softer substrates are more likely to develop into neuronal cells [28]. Several studies have reported the incorporation of various biomaterials into GelMA to optimize its mechanical limitation [29, 30]. In our study, the addition of MWCNTs/Co to the GelMA hydrogel significantly improved its mechanical strength, which might be due to hydrogen bonding interactions between the MWCNTs/Co and the GelMA polymer. This enhancement offers promising potential for surgical applications. Furthermore, it is essential to achieve a balance between the degradation rate and the structural integrity necessary for cell growth. The enzyme-mediated degradation profiles indicated that GelMA–MWCNTs/Co hydrogel exhibited a reduced degradation rate, which may be attributed to the reinforcement of internal structural networks by the MWCNTs/Co nanocomposites. Subsequent proliferation assays also demonstrated that the presence of MWCNTs/Co in the GelMA matrix provided a conducive environment that favors cellular growth and proliferation (Fig. 2C), which is critical for the successful application of these materials in vascularization and tissue engineering.

Hypoxia-induced growth factors are crucial drivers of vascular development, both during embryonic stages and postnatal vascular remodeling [31, 32]. Under hypoxic conditions or hypoxia-mimicking environments induced by specific factors, the activity of prolyl hydroxylase domain (PHD) enzymes is inhibited, leading to the stabilization and accumulation of HIF-α isoforms (HIF-1α, HIF-2α, and HIF-3α) in the cytoplasm [33]. Subsequently, HIF-1α translocated to the nucleus forms a complex with HIF-1β. This HIF-1 complex functions collaboratively to activate the transcription of various genes involved in the cellular response to hypoxia, including cytokines, chemokines, growth factors, and transcription factors [34], which play pivotal roles in promoting neovascularization and tissue regeneration. Among these factors, VEGF stands out as the primary driving force behind hypoxia-induced vascular formation [35, 36]. Our data supported the hypothesis that the GelMA–MWCNTs/Co hydrogel could mimic a hypoxic microenvironment conducive to vascularization by activating the HIF-1α pathway. As VEGF is a crucial proangiogenic factor secreted by cells that plays a vital role in endothelial cell function and vascularization [37], the significant upregulation of VEGF release provided evidence of the efficacy of hydrogel in initiating hypoxia-induced proangiogenic responses (Fig. 3B-C). The initial surge in VEGF expression followed by a decrease suggested a dynamic process of VEGF utilization for endothelial cell functions, consistent with the cellular behaviors during vascular development and remodeling. Subsequent studies significantly advanced our understanding of the angiogenic behavior of HUVECs and SCAP within a 3D coculture system. The observed vascular formation within the GelMA–MWCNTs/Co hydrogel underscored the importance of a simulated hypoxic environment. The presence of MWCNTs/Co nanocomposites appears to be a determinant factor in promoting vasculogenesis, likely through the modulation of VEGF expression and subsequent enhancement of angiogenic activity. The differentiation of SCAP into vascular smooth muscle cells expressing SM22α and their integration into vessel structures was particularly noteworthy (Fig. 5), implying that SCAP not only contributed to the initial angiogenic events but also played a role in the subsequent stabilization and maturation of the vascular network. The perivascular distribution of SCAP differentiated cells and the formation of luminal spaces provide compelling evidence of the potential for these engineered structures to support blood perfusion post-implantation.

During embryonic development, the formation of both vasculature and the nervous system is governed by overlapping signaling pathways, and their growth is mutually influenced and interconnected [2, 38]. Therefore, simultaneous reconstructing vascularization and innervation are essential for functional tissue regeneration. Based on the bioactivity of cobalt ion-releasing hydrogels, we aimed to develop a cell-loaded scaffold capable of pre-vascularization to facilitate the complex tissue regeneration process of vascularization and innervation. Firstly, we found a synergistic effect of the hypoxia-mimetic environment on the neural differentiation of electrically stimulated iSCAP (Fig. 6A-D). This finding was consistent with some earlier studies that highlighted the crucial role of the hypoxic microenvironment in various aspects of embryonic development, regeneration, and adult cell repair. Numerous researchers have demonstrated the neuroprotective effects of hypoxia and hypoxic preconditioning in different central nervous system disease models [39, 40]. Moreover, these conditions have also been shown to significantly enhance the regenerative capabilities of neural progenitor cells [41, 42]. Secondly, we observed a robust growth of neural differentiation iSCAP and a concurrent increase in vessel formation within the GelMA/Co + ES group, reinforcing the concept of interdependent relationships between neural and vascular components (Fig. 6A-C). Our results align with the existing studies that have demonstrated a bidirectional communication between the vascular and nervous systems [43]. Mukouyama et al. revealed that the disruption of sensory nerves or the absence of Schwann cells around mouse limb skin correlates with abnormal arterial formation. Similarly, mutations that cause disorganized nerves lead to misaligned arteries following the path of the axons, hinting at the influence of neural structures on vascular patterning [44, 45]. These findings strongly suggest that peripheral sensory nerves are critical in guiding arterial differentiation and vascular branching. The aforementioned investigations also demonstrated that neural-derived factors such as C-X-C motif chemokine ligand 12 and VEGF-A are critical for endothelial cell migration and arterial differentiation, respectively [46]. Notably, our study revealed that HUVEC-formed vascular networks exhibited functional connectivity with neural-differentiated iSCAP under hypoxia-mimicking conditions. The GelMA–MWCNTs/Co hydrogel, in conjunction with iSCAP, synergistically enhanced the assembly of vessel-like structures and facilitated collaborative proliferation between neurally differentiated iSCAP and HUVECs. Thirdly, it should be noted the possibility that the observed increase in vasculature within the GelMA/Co + ES group may also contribute to the neural differentiation in iSCAP (Fig. 6A-C). Blood vessels also play a crucial role in nerve formation, as the vascular system provides essential support for nerve regeneration following injury by delivering blood, oxygen, and nutrients to nerve cells. Studies have shown that VEGF possesses direct neurotrophic and neuroprotective properties on various types of nerve cells [47]. Cattin et al. offered valuable insights into the guidance role of blood vessels in Schwann cell migration. Blood vessels served as conduits, guiding Schwann cells to use them as pathways to transport regenerating axons across neural gaps. Importantly, disruption of these neovasculatures significantly impaired the directional guidance of Schwann cells, resulting in impaired nerve repair [48]. This emphasizes the role of vascular-derived growth factors in neural system development, highlighting their importance in neurotrophic processes across various neuronal cell types. In our study, the enhanced expression level of VEGF in the GelMA/Co groups, particularly following ES treatment, underscored its pivotal role in initiating neovascularization. Moreover, the simultaneous elevation of NGF and BDNF expression levels suggested a potential interrelationship between angiogenesis and neural differentiation (Fig. 6E). These findings indicated that the processes of blood vessel development and nerve tissue regeneration are not only interconnected but may also be mutually regulated by VEGF, NGF, and BDNF.

Our in vivo study was consistent with existing literature that pre-vascularized constructs enhanced post-transplantation integration [49]. The superior performance of the GelMA/Co + ES group, both in terms of vascular density and functionality, suggested that the hydrogel scaffold effectively supported the formation of pre-vascularized networks (Fig. 7A-B). These networks facilitate the inosculation process with the host vasculature, which is critical for successful implanted tissue construct integration. Given that the natural inosculation process of two microvascular networks typically requires several hours to days [50, 51], our data verified that the hydrogel provided a beneficial scaffolding effect for pre-vascularized vessels before their anastomosis with the host vasculature. Also, the rapid anastomosis observed may be attributed to the favorable environment of the scaffold for neural cells to survive. The presence of human CD31-positive microvessels and elevated Tuj1-positive cell areas within the MWCNTs/Co-enriched GelMA scaffolds, especially under electrical stimulation, suggests that the scaffold not only supports angiogenesis but also fosters neurogenesis (Fig. 7C-D). Functional tissue regeneration is an intricate process that relies on the simultaneous interaction between angiogenesis and neurogenesis. This dynamic interaction is crucial as it involves the coordinated increase in vascularization, which provides a constant supply of blood and nutrients to support nerve regeneration and guide regenerating axons toward their intended targets. Simultaneously, chemokines secreted by the growing nerves play a dominant role in the remodeling and branching of blood vessels [52]. Our studies demonstrated that the GelMA–MWCNTs/Co hydrogel had dual effects. GelMA–MWCNTs/Co hydrogel triggered SCAP to secrete VEGF, promoting vasculogenesis in HUVECs. The newly formed blood vessels from this process provided essential nutrients and oxygen for the neurogenesis of iSCAP. On the other hand, the neural differentiated iSCAP secreted NGF and BDNF, guiding blood vessel remodeling and branching. These findings supported the interplay between vascularization and innervation in the GelMA–MWCNTs/Co hydrogel promoted in vivo angiogenesis and neurogenesis to enhance functional tissue regeneration.

Overall, we developed a 3D hypoxia-mimicking GelMA–MWCNTs/Co hydrogel and characterized its controlled release of cobalt ions, which can effectively promote vascularization and innervation. This work provides a theoretical foundation and preliminary data for applying hydrogels in integrating vasculogenesis, angiogenesis, and neurogenesis, with considerable implications in stem cell-based tissue regeneration. However, this study has limitations that warrant further consideration. For instance, utilizing a spinal cord injury model would offer a more effective approach to evaluating the functionality of the GelMA–MWCNTs/Co hydrogel. Furthermore, in our tri-culture experiments involving iSCAP, HUVECs, and SCAP to generate neurovascular structures, we observed that the GelMA–MWCNTs/Co hydrogel scaffold enhanced the expression of growth factors and facilitated interactions between vascular and neural-like structures. However, the specific cellular origins of these growth factors remain uncertain due to the difficulty in segregating the co-cultured cell populations. Future studies should focus on elucidating the distinct contributions of each cell type. A more comprehensive understanding of the underlying mechanisms governing the interactions between vascular and neural components is crucial for advancing the integration of vasculogenesis and neurogenesis.

## Conclusions

Hypoxia-mimicking GelMA–MWCNTs/Co hydrogel displayed a controlled release of Co ions and adequate electroconductivity, promoting vascularization and innervation both in vitro and in vivo. The interactions between pre-vascularization and pre-innervation within an engineered tissue construct can foster vasculogenesis, angiogenesis, neurogenesis, and tissue regeneration. The theoretical framework and the results of this study on utilizing GelMA–MWCNTs/Co hydrogel, mimicking hypoxia and with electroconductivity, to facilitate the formation of neurovascular bundles hold significant translational potential for clinically dental stem cell-based tissue regeneration.

## Data Availability

No datasets were generated or analysed during the current study.
